# Treatment and Pharmacology of Anxiety From Classical Antiquity to the Present Day: Methods and Therapeutic Practices

**DOI:** 10.7759/cureus.106621

**Published:** 2026-04-07

**Authors:** Panagiotis Sideris, Spyros N Michaleas, Marianna Karamanou, George N Vlachakis

**Affiliations:** 1 Department of Internal Medicine, 417 Army Share Fund Hospital, Athens, GRC; 2 Communication Laboratory of Science, Technology and Medicine, Hellenic Open University, School of Humanities, Patra, GRC; 3 Department of History of Medicine and Medical Ethics, National and Kapodistrian University of Athens School of Medicine, Athens, GRC

**Keywords:** anxiety disorders, benzodiazepines, cognitive theory, humoral theory, psychopharmacology, psychosomatic medicine, stress physiology

## Abstract

Anxiety has been an integral component of human experience, which has been explained through a variety of medical, philosophical, and cultural models throughout history. This study is a narrative review aiming to trace the historical evolution of the treatment and pharmacology of anxiety from Classical Antiquity to the present day. In ancient Greek times, it was considered a psychosomatic illness, which was treated medically and philosophically. Through the Medieval and Early Modern periods, theological models were also considered, as were humoral models. The development of modern psychiatry and psychopharmacology has also shown a shift towards a more integrated model of treating anxiety. This historical trajectory highlights the continuity between early holistic approaches and contemporary biopsychosocial models of anxiety. Understanding this evolution provides valuable insight into the complexity of anxiety as both a clinical condition and a deeply human experience. This review demonstrates that modern approaches to anxiety are not a rupture from the past but the result of a long-standing convergence of medical, philosophical, and cultural paradigms.

## Introduction and background

Anxiety, or what we retrospectively describe as anxiety or anxiety-like states, holds a prime position in both medical and philosophical literature throughout recorded history. According to the Diagnostic and Statistical Manual of Mental Disorders, Fifth Edition (DSM-5), anxiety is defined as an emotional state characterized by feelings of tension, worried thoughts, and associated physical changes such as increased heart rate [1]. Even though modern psychiatric literature acknowledges it as a separate class of mental health disorders, as evident in contemporary diagnostic systems such as the DSM-5, its understanding has undergone considerable transformation throughout history [1]. From Classical Antiquity to modern times, anxiety has been described using a range of models, such as humoral theory, philosophical ethics, religious beliefs, and contemporary neurobiological theory [2,3].

In ancient Greek medical and philosophical traditions, emotional disorders were closely related to bodily equilibrium and mental assessment, which reflects early psychosomatic models of health and illness [2]. Philosophical schools, such as Stoicism and Epicurean doctrine, developed therapeutic methods for achieving emotional equilibrium, which is analogous to some aspects of modern cognitive therapies [4]. In subsequent periods, models of anxiety have continued to include theological and moral components, as well as medical ones [2].

The development of modern psychiatry and psychopharmacology in the 19th and 20th centuries marked a significant shift in the direction of clinical classification and biological treatments. This marked a significant shift in the development of standardized diagnostic criteria and pharmacological treatments that have continued to influence contemporary treatments for anxiety disorders [1,2].

It should be noted that the term "anxiety" is used in this review as a broad concept rather than a strictly defined diagnostic category, as its meaning has varied significantly across historical periods. Pre-modern descriptions of emotional distress, including humoral dyscrasia, melancholy, and moral or spiritual pain, do not directly correspond to contemporary diagnostic entities such as those defined in the DSM-5.

The purpose of this article is to present a narrative review that traces the development of the treatment and pharmacology of anxiety from Classical Antiquity to the present day, with a particular focus on changes in therapeutic approaches over time. Despite the extensive literature on anxiety from both clinical and historical perspectives, there remains a lack of integrative studies that systematically trace the continuity between ancient medical and philosophical models and contemporary biopsychosocial approaches.

## Review

Methods (narrative review approach)

This study is a narrative historical review and does not follow a systematic review protocol. Sources were identified through targeted searches of major databases, including PubMed and Google Scholar, as well as through consultation of key textbooks, historical monographs, and landmark review articles in the fields of the history of medicine, psychiatry, and psychopharmacology.

The keywords used included "anxiety", "history of anxiety", "humoral theory", "Stoicism and anxiety", "psychopharmacology", and "anxiety treatment". The selection of sources focused on works representing significant historical milestones, influential theoretical models, and widely cited contributions to the understanding and treatment of anxiety.

For contemporary psychiatry and pharmacology, emphasis was placed on groundbreaking developments (e.g., psychoanalysis, stress theory, benzodiazepines, antidepressants), as well as recent review articles addressing emerging therapeutic approaches. Sources were selected based on their relevance, historical significance, and contribution to the interdisciplinary understanding of anxiety.

Ancient period (Classical Antiquity)

In Classical Antiquity, conditions resembling what may be described as anxiety-like states were not considered distinct psychiatric disorders. Rather, the emotional disturbances were viewed in the context of a more general medical and philosophical model, which centered on the close association between the body and the mind [5,6]. The Greeks' view of emotional distress was a combination of physiological imbalances and the impact of external circumstances on the mind. This is one of the earliest forms of the psychosomatic model of the history of medicine, where emotional disturbances were seen to be influenced by the body, the environment, and the mind [7].

**Figure 1:**
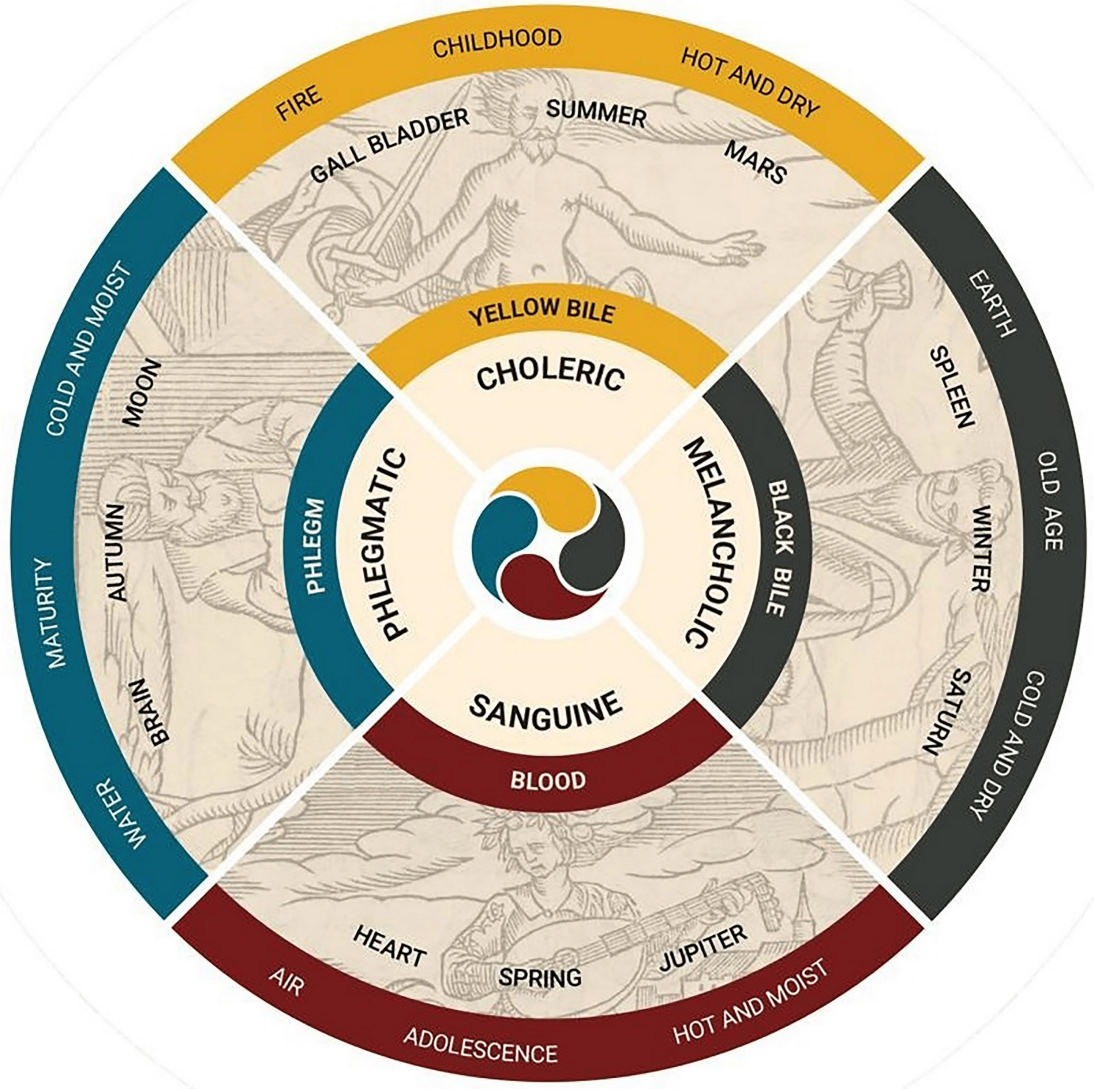
Diagram of the four humors (blood, phlegm, yellow bile, and black bile) as represented in early medical traditions Image Credit: National Library of Medicine (NLM) [10]; reproduced under open-access/public domain conditions

The earliest medical theories of emotional disturbances can be traced back to the school of medicine known as the Hippocratic school, which was established in the fifth and fourth centuries BCE. Hippocratic medicine is associated with the theory of the role of humors in controlling the body and mind of a human being. In other words, the body and mind of a human being are regulated and controlled by the presence of humors. The humors include blood, phlegm, yellow bile, and black bile. The state of health is the harmonious equilibrium of the humors, which is called eucrasia (in Greek: εὐκρασία). On the other hand, the state of illness and emotional disturbances is the state of disequilibrium of the humors, which is called dyscrasia (in Greek: δυσκρασία). It is believed to affect the state of physical health and emotional stability (Figure 1) [8,9].

The physician Galen (129-c. 216 CE), who further elaborated the Hippocratic tradition of medicine, also attempted to establish a more systematic understanding of diseases. In his treatises on diseases and symptoms, he tried to define and classify diseases and symptoms and, at the same time, explore their causes. Galen suggested that emotional disturbances could be caused by the interaction of a person's physical constitution, the functioning of specific organs of the body, and environmental factors. He tried to understand both physical and psychological processes and came up with a comprehensive understanding of how physical and emotional states are connected, thereby providing support to the idea that physical causes may lead to emotional disturbances [11,12]. Although humoral theory lacks empirical validity by modern scientific standards, its emphasis on balance and systemic regulation anticipates contemporary models of homeostasis and psychosomatic interaction.

Galen also looked into the connection between the body and the soul through a type of physiological psychology. He believed that the functions of the soul were related to the body's structure and temperament and that any disruptions to the body's constitution could affect emotions and mental conditions. Therapeutic interventions, as seen in the medical practices of the ancient Greeks, also show an integrated approach to medicine and the understanding of emotions through biological and psychological processes: the use of dietary regimens, lifestyle modifications, and pharmacological interventions to restore physiological balance to the body [13]. Specifically, Galen's pharmacological practice involved the use of complex herbal preparations tailored to the patient's constitution, such as opium-based preparations for sedation, as well as plant-derived substances such as mandrake and henbane, which are believed to relieve agitation and promote sleep. These interventions were often combined with dietary adjustments and environmental modifications, reflecting a comprehensive therapeutic strategy aimed at restoring humoral balance [11,13].

Consistent with such medical theories, philosophical schools also offered a system of concepts to account for the phenomenon of emotional disturbance and psychological well-being. Philosophical contemplation was seen as a therapeutic practice to deal with the phenomenon of emotional disturbances to attain a state of psychological equilibrium. Thus, it was seen as a therapeutic practice to guide an individual to manage the phenomenon of emotional disturbances [14].

Among the philosophical schools of antiquity, the Stoics provided one of the most detailed accounts of emotional disturbance. The Stoic philosophers saw emotions as "cognitive evaluations of the world in terms of what is beneficial or harmful". Chrysippus of Soli (c. 280-c. 206 BCE), the prominent philosopher of the school, saw emotions as "the product of judgment or evaluation concerning external events and the appropriateness of the response to these events". Emotional disturbances, such as fear or anxiety, can thus be reduced through rational reflection and correction of erroneous judgment. The philosophical school of the Stoics thus developed a rational therapeutic approach aimed at achieving apatheia (Greek: ἀπάθεια), understood as the absence of destructive emotional disturbances [6,14].

Epicurean philosophy provided a different yet complementary way to achieve emotional calmness. Epicurus (341-270 BCE) believed that "human beings suffer from anxiety caused by irrational fears such as death and God's wrath". Epicurean ethics hold that the ultimate goal in human life is to achieve a state called ataraxia (Greek: ἀταραξία), which is defined as mental tranquility or absence of mental disturbances. To achieve this state of mind, it is necessary that a person obtain a philosophical understanding of nature by removing ideas that cause fear and anxiety. Intellectual reflections, control, and superstitions are important elements in this philosophy to achieve mental well-being [6,7]. These philosophical approaches can be considered early cognitive frameworks, which show remarkable similarities to modern cognitive-behavioral therapy, particularly in their emphasis on the role of judgment and rational reinterpretation in the regulation of emotional distress [4,15]. These similarities should be understood as analogical rather than indicative of a direct historical continuity or theoretical lineage. However, unlike modern therapeutic models, these approaches were embedded within ethical systems rather than clinical practice.

Collectively, the medical and philosophical ideas of Classical Antiquity provided a series of approaches that are supportive of each other for the comprehension of the problem of emotional disturbance. On the one hand, the medical ideas of Hippocrates and Galen highlighted the significance of physiological equilibrium and regulation. On the other hand, the various philosophical ideas of Classical Antiquity, such as the ideas of the Stoics and the Epicureans, highlighted the cognitive and ethical approaches for the attainment of emotional stability. The early ideas may be considered as the foundation for the subsequent development of the ideas of the philosophy of emotions, psychosomatic medicine, and the development of the treatment of anxiety [7]. In this sense, these early models can also be interpreted as precursors of the modern biopsychosocial approach, as they integrate the physical, environmental, and cognitive dimensions of emotional experience.

Medieval period

The transition from Classical Antiquity to Late Antiquity and the Medieval period was accompanied by a significant transformation in the understanding of emotional disturbances. Whereas Greco-Roman medical and philosophical traditions were grounded in naturalistic and rational explanatory models, later interpretations increasingly framed such conditions within religious and theological contexts. Within this new paradigm, experiences comparable to anxiety-like states were no longer viewed solely as physiological or psychological states but also as expressions of moral conflict and manifestations of divine agency [7,16].

However, it must be noted that despite these developments, Galenic medicine continued in Late Antiquity and in the Middle Ages. In fact, it is certain that medicine continued to be largely associated with humoral theory and that emotional disturbances continued to be associated with imbalances in bodily fluids and temperaments. The application of various forms of therapy such as dietetics, regulation of lifestyle, and use of herbs continued to be associated with the link between physical and emotional well-being that had been established in Classical Antiquity [8].

At the same time, early Christian thought introduced new interpretative frameworks for emotional distress. Influenced in part by Stoic philosophy, early Christian writers adapted the concept of emotional regulation into a moral and spiritual context. Emotional disturbances were often understood in relation to sin, temptation, and the struggle for spiritual purity. The Stoic notion of controlling emotional reactions through rational judgment was transformed into the idea of resisting sinful thoughts and maintaining spiritual discipline [14].

During this period, the concept of emotional disturbances started to blend with theological concepts. Anxiety states could be seen as a state of guilt, fear of God's punishment, or insecurity in faith. However, this does not imply that the medical approach was replaced by the theological approach, but rather both coexisted. This is seen as a reflection of the interface between religion and medicine during this period [7]. This dual framework introduced a productive tension between spiritual and physiological interpretations of emotional distress, rather than a simple replacement of one model by another.

The Renaissance period saw a gradual return to classical sources and naturalistic explanations of human physiology and psychology. Scholars went back to the works of Hippocrates and Galen but also made new observations in anatomy and science. Emotional disturbances started to be understood in relation to emerging medical science, although the role of humors was significant in this regard [16].

One of the most important aspects of the Early Modern period was the development of detailed definitions of melancholia (Greek: μελαγχολία), a term used to describe a broad range of emotional disorders, including what would be described today as anxiety. The work of Robert Burton (1577-1640) on "Anatomy of Melancholy" in 1621 offered an extensive study of the causes and symptoms of melancholia and its various treatments. It was a medical, philosophical, and literary study. It discussed both physiological and psychological aspects of melancholia. This was an example of the continued interconnection between body and mind [17]. At the same time, the persistence of humoral explanations illustrates the gradual and uneven transition toward empirical medical models.

In this era, therapeutic practices were mainly holistic, such as diet, exercise, rest, environmental influences, and psychological and moral treatments, including guidance on ethical conduct, regulation of passions, spiritual discipline, and the cultivation of virtues aimed at achieving emotional balance. This can be said to be a link to the past and the recent concepts of mental health [2,3].

Generally, this period, which began with the end of the ancient era and the start of the modern era, was characterized by continuity and change regarding the management and understanding of emotional disturbances. This period saw the end of the humoral and psychosomatic views, while new religious, moral, and intellectual views were developed and contributed to the change in the management and understanding of anxiety states. This was the platform for the development of modern psychiatry, which later developed into the scientific and clinical management of anxiety states in the 19th and 20th centuries [7].

19th-20th-century psychiatry

The advent of modern psychiatry in the 19th and 20th centuries signaled a major turning point in the understanding and management of emotional disorders, including those that might be termed anxiety-related. This is in direct contradistinction to earlier periods, in which understanding of emotional states was governed by humoral or philosophical constructs, whereas in the modern era, a clinical and scientific approach, as exemplified by observation, classification, and treatment, began to dominate the field of psychiatry. That process led to a progression that is inextricably linked with the advent of institutional psychiatry and the eventual establishment of standardized classification systems, such as the current DSM-5, in which anxiety disorders are recognized as discrete clinical entities [1,18,19].

The Diagnostic and Statistical Manual of Mental Disorders (DSM), first published by the American Psychiatric Association in 1952, represents a significant effort to standardize psychiatric diagnosis through functional criteria. Its current edition, DSM-5-TR, classifies anxiety disorders as a distinct group of conditions characterized by excessive fear, anxiety, and related behavioral disturbances. According to the DSM-5-TR, anxiety is typically defined by symptoms such as persistent and excessive worry, restlessness, muscle tension, sleep disturbances, and impaired functioning.

The main categories of anxiety disorders include generalized anxiety disorder, panic disorder, social anxiety disorder, specific phobias, agoraphobia, and separation anxiety disorder. Each of these conditions is defined by specific diagnostic criteria regarding duration, symptom profile, and functional impairment, reflecting an effort to distinguish pathological anxiety from normal emotional reactions. 

However, while modern diagnostic systems provide greater clinical clarity and standardization, their categories do not directly correspond to earlier conceptualizations of emotional distress. Pre-modern concepts such as humoral imbalance, melancholy, or moral and spiritual distress encompassed a broader and more fluid spectrum of experiences that cannot easily be reduced to distinct diagnostic entities. In this sense, modern classifications represent not only scientific progress but also a transformation in the conceptual understanding of anxiety, from a holistic and context-dependent phenomenon to a more narrowly defined clinical condition.

**Figure 2:**
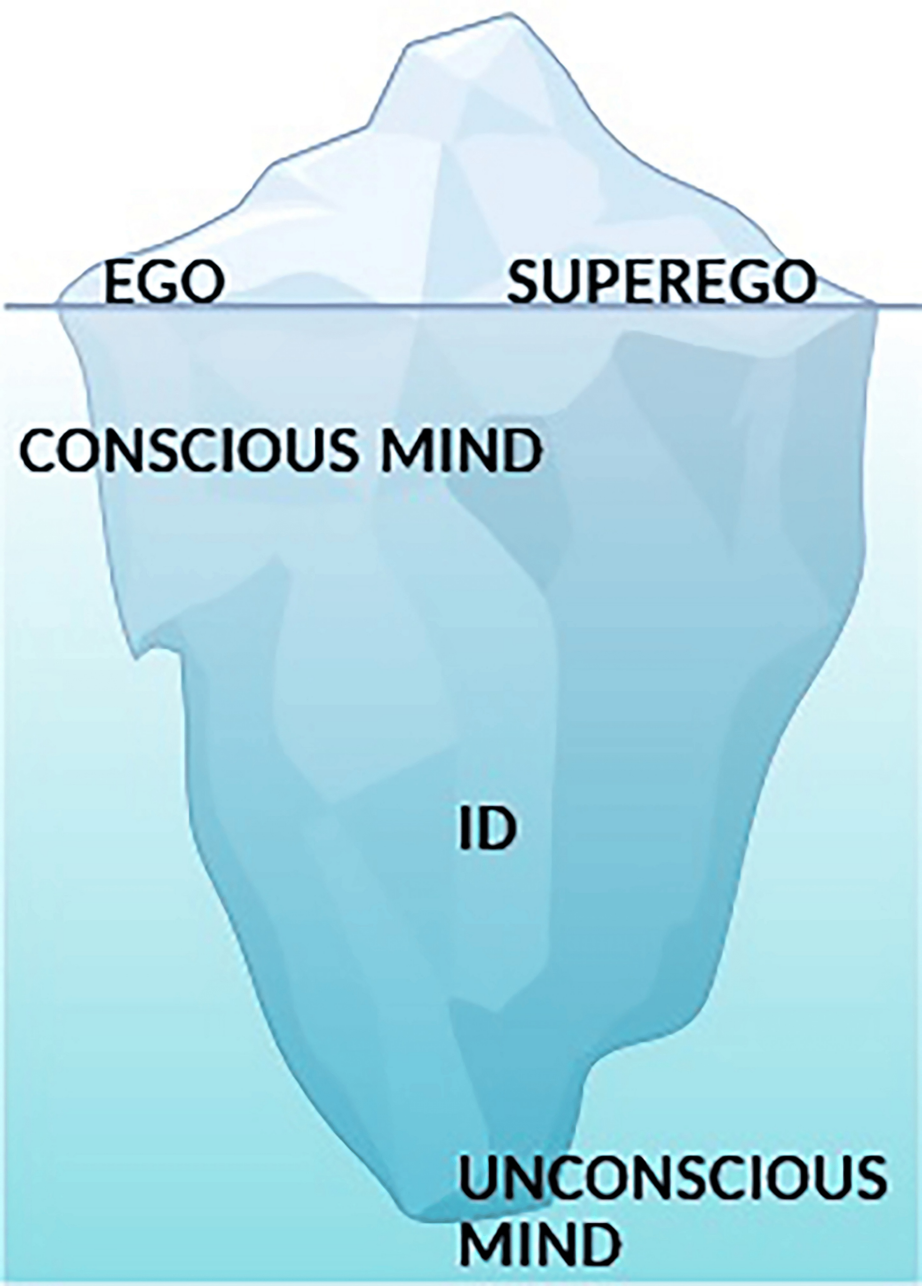
Schematic representation of Sigmund Freud's iceberg model of the mind, illustrating the relationship between conscious and unconscious processes, as well as the structural components of the psyche (id, ego, and superego) The figure illustrates Freud's structural model of the psyche, which is usually depicted as an iceberg, in which only a small portion of mental activity is conscious, while the majority remains unconscious. The id, which resides entirely in the unconscious, represents instinctual drives and desires governed by the pleasure principle. The ego, which operates on both conscious and unconscious levels, mediates between these impulses and external reality, striving to maintain balance. The superego, which represents internalized moral standards and social norms, exerts pressure on the ego to conform to moral expectations. Anxiety arises when the ego perceives a threat arising either from its own instinctual demands, from the superego's prohibitions, or from external reality, thus acting as a signal that activates defense mechanisms to maintain psychological stability. Image Credit: Authors using BioRender.com, in accordance with the platform's license terms [23]

A major milestone in the history of psychiatry during the Early Modern period was the rise of psychosomatic medicine, which reunited the body with the mind from a scientific point of view. The emphasis of Johann Christian August Heinroth (1773-1843) on the significance of psychological and moral factors in illness resulted in a holistic understanding of illness and health. This was reinforced with the rise of psychoanalysis by Sigmund Freud (1856-1939), which provided a scientific understanding of emotional illness in relation to unconscious factors. Thus, anxiety was seen as a result of inner conflicts, especially between the ego and the id (Figure 2) [18,20-22]. 

According to Freud, anxiety arises from conflicts between the id, which represents instinctual impulses and desires, and the ego, which seeks to mediate these impulses in accordance with reality and social norms. When unacceptable impulses from the id threaten to surface into conscious awareness, the ego generates anxiety as a warning signal, activating defense mechanisms such as repression, denial, or displacement, in order to maintain psychological balance. In this context, anxiety is not merely a symptom, but a fundamental regulatory mechanism that reflects the ongoing tension between unconscious desires, the moral constraints imposed by the superego, and the individual's ability to cope with these internal demands.

The theory of stress, which appeared in the 20th century, can be considered a further step in the scientific study of the phenomenon of anxiety. The theory of the general adaptation syndrome, which was put forward by Hans Selye (1901-1982), determined the phenomenon of stress as the physiological response of the organism to the demands of the environment. Selye described the process of the development of the phenomenon of stress in the following stages: (1) the alarm stage, (2) the resistance stage, and (3) the exhaustion stage. This theory helped to understand the relation between the phenomenon of stress and the development of diseases from the biological point of view. It also served as the basis for further study in the field of neurobiology and psychophysiology [24]. Although it has been widely influential, Selye's model has been criticized for its primarily biological focus, which underestimates the role of individual perception and social context in the experience of anxiety.

Following Selye's theory, other researchers like Richard S. Lazarus (1922-2002) and Susan Folkman (1944-2018) focused on the cognitive component of stress. They developed the transactional model, which stated that stress can also be influenced by the individual's perception of the stimuli and their ability to cope with the stimuli. In this theory, it is believed that when an individual perceives the stimuli as harmful and beyond their coping capacity, they develop anxiety. This cognitive theory of stress has had a major impact on modern-day psychological and psychiatric theories [25]. This shift towards cognitive and physiological interpretations reflects a partial return to older holistic approaches, although now based on empirical and experimental methodologies.

There was a tremendous change in the management and treatment of anxiety disorders in the mid-20th century, specifically in the introduction of psychopharmacology. In the beginning, the management of anxiety disorders through pharmacology was carried out using barbiturates, which was deemed effective in the management and treatment of anxiety disorders. However, the abuse and toxicity of the use of barbiturates in the management of anxiety disorders prompted a need to explore other management options that were less abusive [26]. Barbiturates exert their effects by potentiating the inhibitory neurotransmission mediated by gamma-aminobutyric acid (GABA), leading to sedation and anxiolysis. However, their narrow therapeutic index, high risk of respiratory depression, and potential for fatal overdose have significantly limited their long-term clinical use, particularly in the management of anxiety disorders.

An important milestone in the pharmacological management of anxiety disorders has been reached with the advent of benzodiazepines in the 1950s and 1960s, which have shown satisfactory anxiolytic activity along with a slightly improved safety profile, and the outcome has been satisfactory. However, the drawbacks of the use of these drugs, which include the possibility of the development of tolerance, dependency, and withdrawal symptoms, have also shown the limitation of these drugs. It has been seen that these drugs have a place in the management of anxiety disorders, although it is only a matter of a short term [20,26]. This limitation highlights the broader challenge of relying exclusively on pharmacological interventions for complex emotional disorders. Benzodiazepines work by enhancing the activity of GABA-A receptors, resulting in anxiolytic, sedative, and muscle-relaxing effects. Their introduction represented a significant advance over barbiturates due to their broader therapeutic index and lower risk of fatal overdose. Nevertheless, subsequent clinical experience revealed significant limitations, such as tolerance, dependence, withdrawal symptoms, and cognitive side effects, which have restricted their use primarily to short-term or adjunctive therapy.

At the same time, the development of antidepressant medications, including tricyclic antidepressants and later selective serotonin reuptake inhibitors (SSRIs), expanded the therapeutic options for anxiety disorders. These agents were found to be effective not only in treating depression but also in managing various forms of anxiety, thereby contributing to the reconceptualization of anxiety disorders within a neurochemical framework. Modern psychopharmacology increasingly focuses on the role of neurotransmitters such as serotonin, norepinephrine, and GABA in the regulation of mood and anxiety, reflecting a shift toward a neurobiological understanding of emotional disturbances [20,26]. SSRIs work by increasing serotonin levels at synapses and are currently considered first-line pharmacological treatments for many anxiety disorders, including generalized anxiety disorder, panic disorder, and social anxiety disorder. Serotonin-norepinephrine reuptake inhibitors (SNRIs) are also widely used. Compared to benzodiazepines, these agents have a more favorable long-term safety profile, although they may be associated with side effects such as gastrointestinal disturbances, sleep disturbances, and sexual dysfunction. It is important to note that treatment response varies among individuals and across specific anxiety disorders, underscoring the need for personalized approaches. Current treatment approaches emphasize a combination of pharmacotherapy and psychotherapy, particularly cognitive-behavioral therapy, with benzodiazepines typically intended for short-term or adjunctive use rather than as first-line treatment.

Thus, it can be said that the development of modern psychiatry and psychopharmacology reflects a continuum wherein there was an attempt to bring together biological, psychological, and social factors in the explanation of human emotional disturbances. From the psychosomatic approaches of Heinroth and Freud in the earlier days to the present-day biological and psychopharmacological approaches in the 20th and 21st centuries, the human emotion of anxiety has come to reflect a multidimensional construct. The practice of modern-day psychiatry reflects a testament to the multidimensional nature of anxiety as a human emotion and behavioral construct (Table 1) [2,3].

**Table 1:** Historical evolution of anxiety: key models and therapeutic approaches

Period	Conceptual model	Therapeutic approach	Relevance to modern practice
Classical Antiquity	Humoral theory, psychosomatic balance	Diet, lifestyle regulation, philosophical therapy	Early biopsychosocial thinking
Hellenistic Philosophy	Cognitive-ethical models (Stoicism, Epicureanism)	Rational reflection, emotional regulation	Foundations of cognitive-behavioral therapy
Middle Ages	Theological and moral interpretations	Spiritual practices, moral discipline	Integration of belief systems in mental health
Renaissance/Early Modern	Melancholia and humoral persistence	Holistic care (diet, environment, rest)	Transition toward empirical observation
19th to early 20th century	Psychoanalytic and psychosomatic models	Psychoanalysis, moral and psychological approaches	Mind-body integration
Mid to late 20th century	Biological psychiatry, stress theory	Pharmacotherapy (barbiturates, benzodiazepines)	Neurobiological models of anxiety
Contemporary era	Biopsychosocial model	Psychotherapy + pharmacology (selective serotonin reuptake inhibitors, emerging treatments)	Integrated, multidisciplinary care

Despite advances in modern psychiatry, the classification and diagnosis of anxiety disorders remain subjects of ongoing debate. Modern systems such as the DSM-5 have contributed to standardization. However, they have also been criticized for potential overmedicalization and for reflecting culturally and historically dependent frameworks rather than universal categories. The boundaries between normal emotional reactions and pathological anxiety are not always clearly defined, raising questions about the extent to which diagnostic categories capture the complexity of human experience.

Similarly, while psychopharmacological treatments have significantly improved the management of anxiety disorders, they are not without limitations. Variability in patient response, the risk of dependence (particularly with benzodiazepines), and concerns about long-term efficacy highlight the challenges of approaches focused on pharmacotherapy. These limitations underscore the continuing importance of psychotherapy and integrated treatment models, reinforcing the importance of a biopsychosocial perspective in contemporary mental health care.

Beyond pharmacological approaches, modern anxiety management places significant emphasis on psychotherapeutic and holistic interventions. Cognitive-behavioral therapy remains one of the most empirically supported therapies, either as a standalone approach or in combination with pharmacotherapy. Other methods, such as mindfulness-based interventions and acceptance and commitment therapy (ACT), further contribute to the diversification of therapeutic strategies. Compared to pharmacotherapy, these approaches often address underlying cognitive and behavioral mechanisms and can provide more sustainable long-term benefits, although their effectiveness depends on patient engagement and accessibility.

Recent developments in the field include digital mental health interventions, online cognitive-behavioral therapy, and emerging integrated approaches that combine pharmacological and psychological therapies. These trends reflect a broader shift toward personalized and patient-centered care. The findings of this review underscore the importance of maintaining a holistic perspective in the treatment of anxiety, while future research should focus on the interaction between biological, psychological, and social factors, as well as on the development of more tailored and culturally sensitive treatment models.

## Conclusions

This historical analysis underscores the importance of avoiding retrospective diagnostic interpretations and highlights the need to interpret past conceptualizations of emotional distress within their own cultural and epistemological contexts. The history of anxiety is a clear indication of the degree to which this human experience has been influenced by different medical, philosophical, and cultural perceptions. From the holistic approaches of Classical Antiquity, through the religious perceptions of the Middle Ages, and finally, from the scientific discoveries of modern psychiatry, each period has contributed to the overall understanding of emotional suffering. As modern medicine attempts to understand anxiety in terms of diagnostic and pharmacological interventions, it is also understood as a complex experience influenced by a range of different factors. This historical trajectory underscores the necessity of integrative approaches that move beyond reductionist interpretations of anxiety. This is a clear indication of the fact that, despite the discoveries of science, anxiety is as much a human experience as it is a medical phenomenon, and as such, it is a human experience which needs to be understood in a holistic manner. Understanding this historical development can make a significant contribution to the design of more holistic and effective therapeutic approaches to anxiety in the modern clinical setting. At the same time, the importance of an interdisciplinary approach is highlighted, in which medicine, philosophy, and social sciences work together to foster a more comprehensive understanding of mental phenomena.

## References

[1] (2013). Diagnostic and Statistical Manual of Mental Disorders. 5th ed. DSM-5. Diagnostic and Statistical Manual of Mental Disorders, Fifth Edition.

[2] Crocq MA (2015). A history of anxiety: from Hippocrates to DSM. Dialogues Clin Neurosci.

[3] Horwitz AV (2013). Anxiety: A Short History. https://muse.jhu.edu/book/26766.

[4] Nussbaum MC (1994). The Therapy of Desire: Theory and Practice in Hellenistic Ethics. https://toleratedindividuality.wordpress.com/wp-content/uploads/2015/10/therapy-of-desire-theory-and-practice-in-hellenistic-ethics.pdf.

[5] Thumiger C, Singer PN (2018). Mental Illness in Ancient Medicine: From Celsus to Paul of Aegina. Mental Illness in Ancient Medicine: From Celsus to Paul of Aegina. Leiden: Brill.

[6] Ahonen M (2014). Mental Disorders in Ancient Philosophy.

[7] Sideris P (2016). The "Psychosomatic Factor" in Etiology and Treating of Diseases in Hellenic and European Medicine of 19th and 20th Century [in Greek]. Athens: Papazisis Publications.

[8] Nutton V (2024). Ancient Medicine.

[9] Marketos S (2000). History of Medicine [in Greek]. Athens: ZITA.

[10] (2026). "And there's the humor of it": Shakespeare and the Four Humors. https://www.nlm.nih.gov/exhibition/shakespeare-and-the-four-humors/index.html.

[11] (2006). Galen: On Diseases and Symptoms. https://www.cambridge.org/core/books/galen-on-diseases-and-symptoms/FA9FBFC805C9DE8FE095666F743CC51D.

[12] Mattern SP (2013). The Prince of Medicine: Galen in the Roman World. https://librarysearch.mtroyal.ca/discovery/fulldisplay?docid=alma9923182019104656&context=L&vid=01MTROYAL_INST:02MTROYAL_INST&lang=en&adaptor=Local%20Search%20Engine&tab=MRULibraryResources&query=sub,exact,Galen&offset=0.

[13] Hankinson RJ (1991). Galen's anatomy of the soul. Phronesis.

[14] Sorabji R (2000). Emotion and Peace of Mind: From Stoic Agitation to Christian Temptation. https://global.oup.com/academic/product/emotion-and-peace-of-mind-9780199256600?cc=gr&lang=en&.

[15] Beck AT (1979). Cognitive Therapy and the Emotional Disorders. https://archive.org/details/cognitivetherapy0000beck_e3y7.

[16] Greenblatt S (2011). The Swerve: How the World Became Modern. https://wwnorton.com/books/The-Swerve/.

[17] Burton R (1883 (originally published 1621)). The Anatomy of Melancholy. https://dn710606.ca.archive.org/0/items/anatomyofmelanch00burt/anatomyofmelanch00burt.pdf.

[18] Shorter E (1997). A History of Psychiatry: From the Era of the Asylum to the Age of Prozac. https://www.wiley.com/en-ie/A+History+of+Psychiatry%3A+From+the+Era+of+the+Asylum+to+the+Age+of+Prozac-p-9780471245315.

[19] First MB, Caban DN, Lewis-Fernández R (2010). Development of the nosology of anxiety disorders. Anxiety Disorders.

[20] Kaplan HI, Sadock BJ, Sadock VA (2021). Kaplan & Sadock's Synopsis of Psychiatry. Kaplan & Sadock’s Synopsis of Psychiatry. 12th ed..

[21] Christodoulou GN (2012). Psychiatry [in Greek]. et al. Psychiatry. Athens: VITA.

[22] Manos N (1997). Basic Elements of Clinical Psychiatry [in Greek]. Thessaloniki: University Studio Press.

[23] (2026). BioRender. https://biorender.com.

[24] Selye H (1978). The Stress of Life. https://raw.githubusercontent.com/peatysharing/bibliography/main/Hans%20Selye/1978%20-%20Hans%20Selye%20-%20The%20Stress%20Of%20Life.pdf.

[25] Lazarus RS, Folkman S (1984). Stress, Appraisal, and Coping. https://books.google.com.ph/books/about/Stress_Appraisal_and_Coping.html?id=i-ySQQuUpr8C&redir_esc=y.

[26] Healy D (2002). The Creation of Psychopharmacology. The Creation of Psychopharmacology.

